# Auto Deep Spiking Neural Network Design Based on an Evolutionary Membrane Algorithm

**DOI:** 10.3390/biomimetics10080514

**Published:** 2025-08-06

**Authors:** Chuang Liu, Haojie Wang

**Affiliations:** School of Intelligent Science and Information Engineering, Shenyang University, Shenyang 110044, China

**Keywords:** spiking neural network, automatic network design, neural architecture search, evolutionary membrane algorithm, deep learning, deep spiking neural network, image classification

## Abstract

In scientific research and engineering practice, the design of deep spiking neural network (DSNN) architectures remains a complex task that heavily relies on the expertise and experience of professionals. These architectures often require repeated adjustments and modifications based on factors such as the DSNN’s performance, resulting in significant consumption of human and hardware resources. To address these challenges, this paper proposes an innovative evolutionary membrane algorithm for optimizing DSNN architectures. This algorithm automates the construction and design of promising network models, thereby reducing reliance on manual tuning. More specifically, the architecture of DSNN is transformed into the search space of the proposed evolutionary membrane algorithm. The proposed algorithm thoroughly explores the impact of hyperparameters, such as the candidate operation blocks of DSNN, to identify optimal configurations. Additionally, an early stopping strategy is adopted in the performance evaluation phase to mitigate the time loss caused by objective evaluations, further enhancing efficiency. The optimal models identified by the proposed algorithm were evaluated on the CIFAR-10 and CIFAR-100 datasets. The experimental results demonstrate the effectiveness of the proposed algorithm, showing significant improvements in accuracy compared to the existing state-of-the-art methods. This work highlights the potential of evolutionary membrane algorithms to streamline the design and optimization of DSNN architectures, offering a novel and efficient approach to address the challenges in the applications of automated parameter optimization for DSNN.

## 1. Introduction

Deep neural networks (DNNs), as the cornerstone of modern artificial intelligence, have achieved transformative breakthroughs in fields such as computer vision, natural language processing, and speech recognition [1,2,3,4]. These networks leverage a hierarchical architecture inspired by biological neural systems, enabling the extraction and representation of multiscale features across different abstraction levels where lower layers specialize in low-dimensional, local feature extraction, while higher layers focus on high-dimensional semantic representations [5,6]. The training paradigm of DNNs is fundamentally driven by the error backpropagation (BP) algorithm, which constructs an end-to-end optimization framework [7]. Through the chain rule, BP enables efficient gradient-based updates across all network layers, facilitating the joint optimization of large-scale neural architectures [8,9].

However, the deployment of such advanced architectures remains constrained by significant technical limitations [10,11,12,13]. Their performance critically depends on three key factors:

- The availability of massive, high-quality labeled datasets;
- Access to specialized hardware accelerators (e.g., GPU clusters and computational servers);
- Substantial energy resources and thermal management systems.

These computational and energetic demands create substantial barriers, limiting the practical development of DNNs on edge computing devices such as wearable devices, mobile phones, and unmanned aerial vehicles (UAVs) [12,14]. Current hardware platforms are constrained by limited power budgets and computational capacities, rendering them incapable of sustaining the full inference workload of DNN models. This disparity highlights a critical adaptability challenge for deep learning technologies in embedded and resource-constrained environments [15,16].

Spiking neural networks (SNNs), inspired by the biological neural networks in the human brain, represent a biologically inspired computational paradigm that models neural activity at the single-neuron level. This approach offers both the potential for achieving higher cognitive intelligence and the capability for ultra-low-power neuromorphic computation through event-driven, spike-based processing [16,17]. Unlike traditional DNNs, SNNs incorporate biophysically plausible mechanisms, such as temporal dynamics in spike signal processing, enabling them to encode and transmit information through multi-dimensional spike trains. This unique characteristic allows SNNs to capture richer neural representations, including critical features like neural oscillations and spike phase information, which are fundamentally inaccessible to traditional rate-based DNNs.

DNNs and SNNs exhibit distinct characteristics across various key features [18]. DNNs utilize artificial neurons that compute weighted sums and apply activation functions, whereas SNNs mimic biological neurons through spiking events and threshold-based firing mechanisms [19]. DNNs process inputs statically without explicit temporal dynamics, while SNNs inherently handle temporal information via spike timing and event-driven processing. In terms of energy efficiency, DNNs typically demand high computational resources and energy, especially when deployed on GPUs or CPUs, whereas SNNs are more energy-efficient due to their sparse and event-driven nature [20]. Training DNNs relies on well-established backpropagation and gradient descent methods, albeit with significant computational intensity. In contrast, training of SNNs is more complex due to the non-differentiable nature of spikes, often necessitating surrogate gradients [21]. Architecturally, DNNs commonly employ dense, convolutional, and recurrent layers, while SNNs leverage specialized architectures that capitalize on spiking dynamics. DNNs are widely applied in image recognition, natural language processing, and reinforcement learning, whereas SNNs excel in processing temporal data, such as gesture recognition, auditory processing, and real-time sensor data [22]. Scalability is a well-established strength of DNNs, which can be scaled to very large models like Transformers and GPT series. For SNNs, scalability remains an active research area with recent progress in scaling up. Regarding biological plausibility, SNNs are more biologically plausible than DNNs, as they closely mimic the spiking behavior and information processing mechanisms found in biological brains [23]. Overall, DNNs offer well-established training methods, broad application domains, and extensive tools and frameworks. However, they face challenges related to high computational and energy costs and limited temporal data processing capabilities. On the other hand, SNNs present advantages in energy efficiency, suitability for temporal data, and biological plausibility but encounter complexities in training, design, and the limited availability of tools and frameworks. A comparison of DNNs and SNNs is summarized in Table 1.

Based on the summary in Table 1, many scholars have begun to explore models that integrate the both DNNs and SNNs. The emerging field of deep spiking neural networks (DSNNs), which integrates the representational power of DNNs with the energy-efficient computation of SNNs, presents a promising pathway toward realizing human-level artificial intelligence. However, the binary nature of spike trains and the non-differentiability of spike-based activation functions present fundamental challenges to the direct application of backpropagation (BP)-based optimization techniques [24]. This methodological bottleneck has led to an open research question regarding the development of effective training methodologies for DSNNs [25].

To address these challenges, researchers have proposed a variety of alternative learning paradigms and training strategies as follows [26].

- DNN-to-SNN Conversion Frameworks
  The first category of training methodologies involves DNN-to-SNN conversion techniques [24], which aim to transfer pre-trained parameters (weights and biases) from conventional artificial neural networks (ANNs) to structurally analogous SNNs. This approach leverages the representational power of high-performing ANNs by approximating their functional behavior in neuromorphic hardware. Despite their theoretical appeal, these methods exhibit systematic performance degradation during conversion due to inherent architectural mismatches between rate-coded ANNs and temporally-coded SNNs. While researchers have proposed mitigation strategies including weight and activation normalization techniques and noise injection for robustness enhancement, complete elimination of accuracy loss remains an open challenge. More critically, such conversion frameworks typically require rate-based encoding schemes to approximate ANN activation distributions. These trade-offs highlight a fundamental limitation of conversion-based approaches their inability to fully exploit the temporal computation paradigm that constitutes SNNs’ primary advantage over traditional ANNs.
- Spike-Timing-Dependent Plasticity (STDP)-Based Learning
  The second category employs STDP [27], a biologically plausible unsupervised synaptic plasticity mechanism observed across various brain regions. Unlike gradient-based methods, STDP enables layer-wise parameter updates in DSNNs by modulating synaptic weights for both error backpropagation and gradient computation of spiking activation functions. While STDP excels at extracting shared statistical features among input samples through its Hebbian learning principle, it inherently struggles with discriminative feature learning due to its symmetric update rule for potentiation and depression. To address this limitation, researchers have proposed several STDP variants that incorporate additional biological mechanisms. These limitations indicate that although STDP-based approaches effectively capture low-level statistical correlations, they currently fail to fully unlock the biologically inspired intelligence potential of SNNs—especially when it comes to high-level cognitive tasks demanding nuanced feature discrimination.
- Direct Training Approaches for Pre-designed DSNNs
  The third category focuses on direct training methodologies for pre-structured DSNNs, with the SpikeProp algorithm and its various enhanced variants representing the most prominent examples [17]. These approaches attempt to overcome the non-differentiability challenge of spiking neurons by leveraging mathematical approximations, such as the linearization assumption in SpikeProp’s gradient estimation framework. While several mitigation strategies have been proposed, including weight regularization constraints, gradient normalization techniques, non-leaky PSP function, and multi-bit spike encoding schemes, these solutions significantly increase computational complexity, rendering them infeasiable for large-scale network implementations.

Within the domain of DSNNs research, these approaches collectively constitute the current cutting-edge of DSNNs exploration. Ongoing investigations are centered on enhancing training stability, optimizing convergence characteristics, and boosting computational efficiency—all while preserving biological plausibility [28,29,30]. In response to the aforementioned issues, this paper proposed a evolutionary membrane algorithm for automatically constructing and designing promising DSNN models. This paper combines the advantage of population diversity in evolutionary membrane algorithms with the issue of the large discrete search space that needs to be addressed and integrates it with a weight-sharing strategy. Building on this, this paper further contemplates the debate on supernet sampling training methods and the contradiction between traditional methods and weight-sharing-based methods in the field of the SNN architecture search. This paper conducts research on the overall network architecture of the DSNNs model from a microscopic to a macroscopic perspective and optimizes the training process of the DSNNs model in terms of the algorithm. Finally, comparisons with manually designed neural networks and the existing state-of-the-art NAS methods demonstrate that this is an effective exploration and attempt. The main contributions of this paper can be summarized as follows.

- For the first time, an evolutionary membrane algorithm is employed for automatically constructing and designing promising DSNN models.
- A search space consisting of the architectures of DSNNs is defined. The proposed algorithm is utilized as a search mechanism to find the best architectures of DSNNs.
- The simulation results verify the effectiveness of the proposed algorithm on the CIFAR-10 and CIFAR-100 datasets.

An overview of the rest of this paper is described below. Section 2 discusses the convolutional neural network, spiking neural network, and neural architecture search. In Section 3, the proposed algorithm is introduced in detail, with particular focus on how to design an evolutionary membrane algorithm to optimize DSNNs. In Section 4, the proposed algorithm is compared with some state-of-the-art algorithms, and the simulation results on the CIFAR-10 and CIFAR-100 datasets are evaluated and discussed. Finally, Section 5 summarizes the conclusions of this paper.

## 2. Related Work

### 2.1. Convolutional Neural Network

In the field of deep learning, Convolutional Neural Networks (CNNs) represent a specialized class of DNNs, with their conceptual origins tracing back to the seminal 1968 work of Hubel and Wiesel [25]. CNNs are predominantly employed for analyzing visual imagery and share architectural similarities with other standard neural network paradigms such as feedforward systems [31]. These networks are fundamentally structured around hierarchically organized neurons, enabling them to autonomously learn hierarchical feature representations, which is a critical ability when extracting and identifying salient patterns within image data [32].

**Figure 1:**
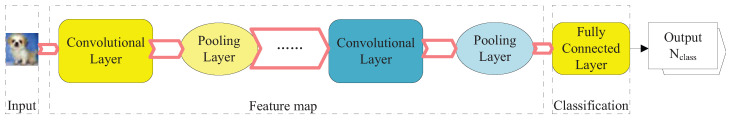
Basic architecture of convolutional neural network.

As illustrated in Figure 1, the CNN architecture maintains consistent three-dimensional tensor representations throughout the input–output pipeline. While convolutional and fully connected layers contain trainable parameters, specialized layers such as activation functions introduce essential nonlinearities without additional parameter overhead. Different layer types exhibit distinct parameter requirements tailored to their computational roles. The following sections systematically examine the CNN’s principal architectural components.

A CNN consists of four fundamental components, detailed as follows.

- Input Layer: Receives raw input data (typically structured as three-dimensional tensors for image-based tasks);
- Feature Map Layer: Performs feature transformation through convolutional operations and nonlinear activations;
- Classfication Layer: Produces classification scores through probabilistic scoring mechanisms;
- Output Layer: Outputs classfication results.

### 2.2. Spiking Neural Network

Spiking Neural Networks (SNNs), as third-generation artificial neural networks, simulate the discrete information transmission mechanism of biological neurons through spike-based signaling [10]. They employ neuron models such as the Leaky Integrate-and-Fire (LIF) and Hodgkin–Huxley models to precisely describe the electrophysiological characteristics of neurons, and they utilize learning rules like spike-timing-dependent plasticity (STDP) to process spatiotemporal information dynamically [33]. SNNs encode information using spike timing, frequency, or phase, and overcome the limitations of backpropagation through training methods such as surrogate gradient, resulting in network architectures including feedforward, recurrent, and hierarchical structures [34]. SNNs have demonstrated advantages in low power consumption and real-time processing in fields such as neuromorphic chips (e.g., IBM TrueNorth, Intel Loihi), robotics control, brain–computer interfaces, and dynamic vision sensor processing. Despite challenges related to hardware compatibility and training efficiency, SNNs are increasingly becoming a core bridge connecting biological neuroscience and artificial intelligence, providing theoretical support and technical pathways for cutting-edge areas such as edge computing and cognitive computing.

At the core of SNNs are sophisticated neuron models that play a crucial role in accurately describing the electrophysiological characteristics of neurons [35]. The Leaky Integrate-and-Fire (LIF) model, for instance, is one of the most commonly used simplified models. It describes how the membrane potential of a neuron changes over time, gradually decaying due to leakage currents, and generates a spike when the input exceeds a certain threshold. This model provides a computationally efficient yet reasonably accurate representation of neuronal behavior, making it suitable for large-scale simulations and practical applications. On the other hand, the Hodgkin–Huxley (HH) model is based on the detailed biophysical properties of ion channels in biological neurons. It offers a high level of biological realism by precisely simulating the complex ion fluxes across the neuronal membrane. However, its computational complexity is relatively high, which limits its application in large-scale networks. To strike a balance between computational efficiency and biological accuracy, the Izhikevich model was developed. It can reproduce a wide range of neuronal firing patterns with relatively low computational cost, making it an attractive choice for simulating large-scale SNNs.

Despite their many advantages, SNNs also face several challenges. One of the main challenges is the training efficiency [10,16]. Although methods like surrogate gradient have been developed to enable training of SNNs, the training process is still more complex and time-consuming than that of traditional artificial neural networks. The non-differentiability of the spike function and the complex dynamics of the neurons make it difficult to optimize the network parameters effectively. Another challenge is hardware compatibility. While neuromorphic chips offer a promising platform for SNNs, the current hardware technology is still in the early stages of development, and there are limitations in terms of scalability, integration, and compatibility with existing software systems. Additionally, the lack of a unified theoretical framework for SNNs is also a significant obstacle. Unlike traditional artificial neural networks, which have a well-established mathematical foundation and a wide range of theoretical tools for analysis and optimization, the theoretical understanding of SNNs is still relatively limited. This makes it difficult to develop general-purpose training algorithms and analyze the performance of SNNs in a systematic way.

### 2.3. Neural Architecture Search

Neural architecture search (NAS) is an automated machine learning (AutoML) technique that aims to discover optimal neural network architectures for specific tasks without manual intervention [12]. Traditional neural network design relies heavily on human expertise, requiring extensive trial-and-error to achieve high performance [36]. NAS automates this process by exploring a vast search space of possible architectures, evaluating their performance, and selecting the best-performing ones [37]. This approach significantly reduces the time and effort required for model development while enabling the discovery of novel architectures that may outperform human-designed models.

**Figure 2:**
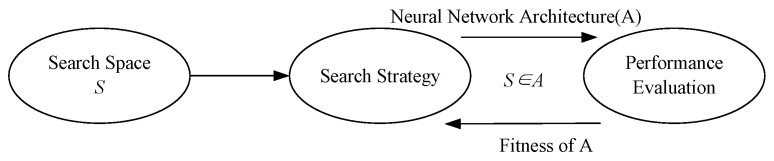
Framework diagram of neural architecture search.

In neural architecture search, the search space, search strategy, and performance evaluation are the three core components, and they are shown in Figure 2. The search space determines “where to search”, the search strategy defines “how to search”, and performance evaluation determines “how to measure the quality of the search results” [26]. By properly designing these three components, the efficiency and effectiveness of NAS can be significantly improved [31]. The search space defines all possible architectures NAS can explore, such as layer types, hyperparameters, and connectivity patterns. The search strategy determines how NAS navigates this space, employing methods like reinforcement learning [38], evolutionary algorithms [39], or gradient-based optimization [40]. Performance estimation evaluates candidate architectures, often using partial training, weight inheritance, or neural predictors to reduce computational costs. Despite its potential, NAS faces challenges such as high computational demands, search space design limitations, and generalization issues across different tasks.

## 3. Proposed Algorithm

In spiking neural architecture search, a supernet is a large-scale network that encompasses all possible architectures within the search space. Its nodes represent computational operations (such as convolution and pooling), and its edges indicate data flow connections. Each layer of a supernet contains multiple candidate operation blocks (such as convolutional layers, pooling layers, and skip connections) [16,41]. The combination of these operation blocks forms a vast discrete search space. For example, assuming the supernet has *L* layers and each layer has *K* types of operation blocks to choose from, the theoretical number of possible sub-networks is $K^{L}$. For a medium-sized supernet, this number can be astronomical. Directly enumerating all possible sub-networks in such a large search space is impractical, hence the need for efficient search methods [42].

**Figure 3:**
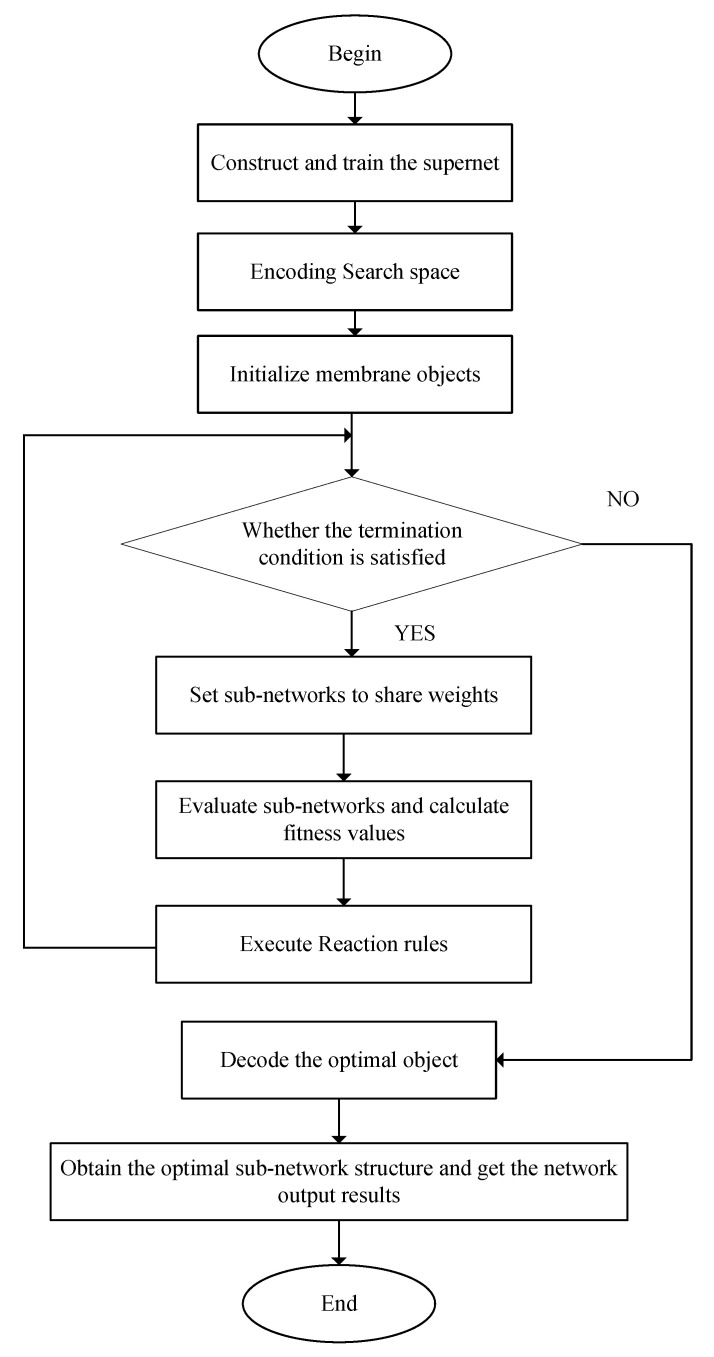
The flowchart of neural architecture search.

Combining the efficient weight-sharing mechanism of the one-shot method with the global optimization capability of evolutionary algorithms enables rapid identification of well-performing sub-network architectures in a large discrete search space. In such a vast discrete search space, to search for the sub-network with the best performance, this paper proposes an evolutionary one-shot neural architecture search method based on membrane computing. The flowchart of the proposed algorithm is shown in Figure 3. The parallelism and flexibility of membrane computing are utilized to divide the search space into multiple sub-regions, each independently undergoing evolutionary search. The selection, crossover, and mutation operations are then repeated to continuously update the population within each membrane. During the iteration, there is communication between membranes to transfer well-performing sub-network architectures or optimization information to other membranes, promoting global optimization. The global and local search coordination is implemented through communication mechanisms between membranes. The search is stopped when a certain number of iterations or convergence conditions are met.

Algorithm 1 illustrates the details of the evolutionary process of the proposed algorithm in the skin membrane. Based on the proposed encoding strategy, the population is randomly initialized in the skin membrane, where each object represents a candidate neural network structure. First, an initial object $P_{0}$ of size *N* is randomly generated using a fixed-length integer encoding strategy (Line 1). The fitnesses of objects $P_{0}$ are calculated according to the accuracy of the neural network (Line 2). Each object in the initial population $P_{0}$ is trained on the training dataset and then evaluated on the valid dataset to obtain the fitness value of each object (Line 3). We determine whether the maximum termination condition (Max_Gen) of the proposed algorithm is met (Line 4). The communication rules are invoked, and the objects are sent to the membranes for evolution (Line 5–6). The objects are evolved in the membrane, and the evaluation results of these objects are returned from the membrane (Line 7). During environmental selection, objects are combined to form a larger population of size 2N. The top *N* objects from different membranes are determined (Line 9). Finally, to reduce unnecessary training generations, an early stopping strategy is adopted, i.e., the loop is terminated when the performance of the neural network no longer shows significant improvement over several generations (Lines 10–12). The final object with the best fitness is determined. The proposed algorithm then outputs this best object and decodes it into the corresponding neural network architecture (Line 14). This optimized neural network is then fully trained and evaluated on the test dataset.
**Algorithm 1** The pseudo-code of the proposed algorithm in the skin membrane.**Input:** Let *N* be the object size, Max_Gen be the number of iterations, *G* be the number of elementary membranes, and *A* be the evolved object set**Output:** Final objects  1:Objects ($P_{0}$) ← Initialization(*N*)  2:Evaluate(Objects)  3:*A*← Objects  4:**for** i=1; i<Max_Gen; i++**do**  5:      **for** j=1; j<G; j++ **do**  6:            Mem(j)← Evolving(Mem(j)) by Invoking communication rule  7:            *A*←A⋃Mem(j)  8:      **end for**  9:      Objects ← A(1:N,:)10:      **if** no longer shows significant improvement **then**11:        break;  12:      **end if**13:**end for**14:Final the bset Object

Algorithm 2 illustrates the details of the evolutionary process of the proposed algorithm in the membrane. The rules of crossover, mutation, and selection are executed in the evolutionary process of the membrane. Subsequently, two parent objects are selected from $P_{t}$ using a binary tournament selection method (Line 1). Two offspring objects are generated by executing the crossover rule (Line 2). Crossover combines the genetic material of two parent objects to produce offspring. This process allows for the mixing of traits from both parents, creating new combinations of genes that may lead to more fit objects. The role of the mutation operation is to introduce genetic diversity into the population by randomly altering some genes of individuals (Line 3). This helps to prevent the population from converging too quickly to a local optimum and maintains the exploration capability of the evolutionary algorithm, thus increasing the probability of finding the global optimum. The fitnesses of objects are calculated according to the accuracy of the neural network (Line 4). The best-performing objects and their fitness values are returned to the skin membrane (Line 5).
**Algorithm 2** The pseudo-code of the proposed algorithm in membranes.**Input:** Let Objects ($P_{i}$) from the skin membrane**Output:** Final Objects  1:Two parent objects are selected from $P_{i}$.  2:Executing the crossover rule  3:Executing the mutation rule  4:Evaluate(Objects)  5:Final Objects

### 3.1. Constructing the Supernet

#### 3.1.1. Supernet Sampling

In the spiking NAS study, the training methods for the supernet are a critical component of the NAS process. Specifically, a one-shot weight-sharing approach is employed for training and sampling the supernet [43]. Weight sharing in NAS is a crucial acceleration strategy. It enables different candidate models to share weights, eliminating the need to train each model from scratch and thereby significantly reducing computational overhead. The core idea of weight sharing is to cover the entire search space through a supernet, where all candidate sub-models share the weights of this supernet. The core ideas behind this method are as follows:

- Weight Sharing: All candidate architectures share the weights of the supernet, which avoids the need to train each architecture individually and significantly reduces computational costs.
- Single-Path Uniform Sampling: During the training of the supernet, architectures are sampled uniformly from the search space. Each time a sub-model is trained, the optimized weights are shared with the supernet, allowing other sub-models to directly inherit these weights for evaluation.

This approach avoids training each sub-model from scratch, significantly reducing training time. This method is simple and effective, ensuring that all candidate architectures are optimized evenly during training.

#### 3.1.2. Training Process of the Supernet

The supernet of spiking NAS is constructed according to the number of layers *L* and the set of candidate operation blocks $\left\{O_{1},O_{2},\ldots ,O_{K}\right\}$ for each layer [44]. We train the supernet using the training dataset to optimize its weights. The weights of the supernet are initialized, which will be shared by all sub-networks. The supernet is trained using the training dataset to optimize its weights. The training objective is to make the supernet perform well across all possible sub-networks. Joint training is typically used, where both the weights of the supernet and the architecture parameters of the sub-networks are optimized simultaneously. Sampling of different sub-network architectures from the supernet is evaluated, inheriting the weights of the supernet to quickly evaluate their performance, thereby identifying the optimal sub-network architecture.

The training process of the supernet in spiking NAS is as follows.

- Supernet Initialization: A supernet that encompasses all candidate architectures is constructed.
- Sampling Training: In each training iteration, an architecture is randomly sampled from the supernet.
- Weight Update: The weights of the sampled architecture are updated, which in turn reflects on the shared weights of the supernet.

### 3.2. Membrane Computing

Membrane computing (P-system) is a computational model inspired by the structure and function of biological cell membranes [45]. It simulates the compartmentalization, communication, and information-processing mechanisms of cell membranes to perform computations [46]. Membrane computing is highly parallel and flexible, capable of handling multiple sub-problems simultaneously and coordinating optimization at different levels. The membrane computing framework divides the search space into multiple sub-regions, each corresponding to a membrane. The number of membranes can be adjusted based on the complexity of the search space and computational resources. Evolutionary search operations (such as selection, crossover, and mutation) are independently conducted within each membrane. The number of membranes can be adjusted based on the complexity of the search space, which is set to 4 in this paper.

Communication rules are designed between membranes to allow information exchange. Communication between membranes allows for better coordination between global and local searches, avoiding local optima. For example, sub-network architectures that perform well in one membrane can be transferred to other membranes, or global optimization information can be fed back to individual membranes. The membrane computing framework provides high flexibility in the search process, allowing easy adjustment of search strategies and parameters to adapt to different search spaces and task requirements.

#### 3.2.1. Initialization of Object

A set of sub-network architectures is randomly generated within each membrane as the initial population. These sub-networks are sampled from the supernet and inherit its weights. The sub-network architectures are evaluated within each membrane using the validation dataset to calculate their performance metrics. The sub-network architectures that perform well based on performance metrics selected as parents for subsequent crossover and mutation operations.

#### 3.2.2. Crossover Rule

Within each membrane, two parent sub-network architectures are randomly selected, and crossover operations are performed on specific layers or modules. The certain operation blocks of one parent network are exchanged with the corresponding operation blocks of another parent network. The purpose of crossover is to generate new sub-network architectures that inherit the strengths of the parents.

#### 3.2.3. Mutation Rule

Random mutations are introduced into parent sub-network architectures by altering the operation block types, connection schemes, or hyperparameters of specific layers, thereby fostering new diversity. Mutation helps to avoid local optima in the search process and explore a broader search space.

#### 3.2.4. Determination of the Optimal Sub-Network

The best-performing sub-network architecture from all membranes is selected as the candidate optimal sub-network. Furthermore, the selected optimal sub-network architecture is trained to fine-tune its inherited weights from the supernet to improve its performance. The final optimized sub-network architecture is validated using an independent test dataset to evaluate its performance in practical tasks.

## 4. Experimental Validation and Result Analysis

### 4.1. Benchmark Datasets and Metrics

The CIFAR-10 and CIFAR-100 datasets represent the most widely adopted benchmark datasets in the research of NAS. On the CIFAR-10/100 datasets, given that their official splits strictly ensure class balance, we adopt the accuracy metric to evaluate model performance. Specifically, the accuracy metric is employed for two compelling reasons.

- Intrinsic balance: Every class receives an identical number of training and test samples, eliminating any majority-class bias by design.
- Community consensus: Pioneering NAS studies, ranging from DARTS and Genetic-CNN to AutoSNN, exclusively utilize accuracy across these benchmarks.

#### 4.1.1. CIFAR-10 Dataset

CIFAR-10, originally compiled and curated by Alex Krizhevsky and Ilya Sutskever under the supervision of Geoffrey Hinton, constitutes a compact yet representative dataset for object classfication tasks. This synthetic dataset comprises 10 distinct classes of RGB color images, including airplane, automobile, bird, cat, deer, dog, frog, horse, ship, and truck.

**Figure 4:**
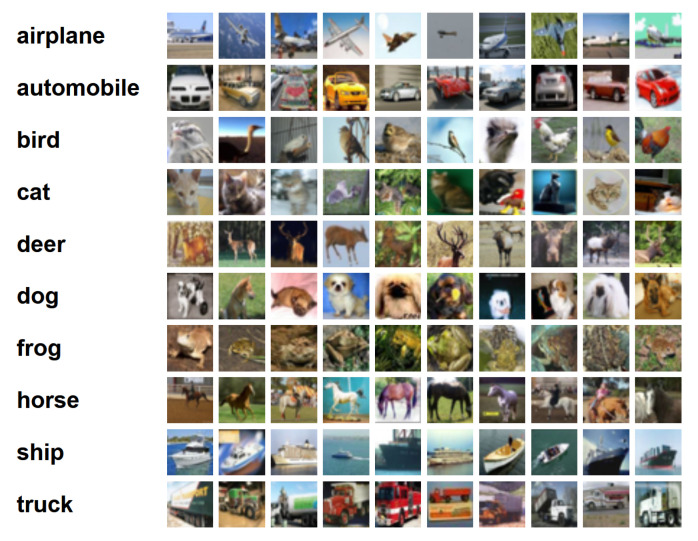
The CIFAR-10 dataset.

With its balanced class distribution and moderate complexity, CIFAR-10 provides a standardized evaluation platform that enables fair comparison across diverse NAS methodologies. The dataset contains 60,000 color images, which are 32*32 in size and divided into 10 classes, with 6000 images in each class. Among them, 50,000 images are used for training, forming 5 training batches, with 10,000 images in each batch; the other 10,000 images are used for testing and form a single batch. The data in the test batch is taken from each of the 10 classes, with 1000 images randomly selected from each class. The remaining images are randomly arranged to form the training batches. The dataset’s relatively small size (50,000 training and 10,000 test images at 32 × 32 resolution) facilitates rapid prototyping while maintaining sufficient complexity to challenge architectural optimization algorithms. Figure 4 shows the classes in the dataset, as well as 10 random images from each.

#### 4.1.2. CIFAR-100 Dataset

As shown in Table 2, the CIFAR-100 dataset represents an extended benchmark counterpart to CIFAR-10, specifically designed to evaluate fine-grained classification capabilities in computer vision research. It has 100 classes, with each class containing 600 images. There are 500 training images and 100 test images in each class. While sharing its fundamental characteristics with CIFAR-10 (32 × 32 RGB images), CIFAR-100 introduces significantly greater classification complexity through its hierarchical label structure. There are 500 training images and 100 testing images per class. The 100 classes in the CIFAR-100 are grouped into 20 superclasses. Each image comes with a ’fine’ label (the class to which it belongs) and a ’coarse’ label (the superclass to which it belongs).

This hierarchical annotation scheme enables simultaneous evaluation of both fine-grained and coarse-grained classification performance, making CIFAR-100 particularly valuable for studying multi-level representation learning. The increased number of classes and reduced intra-class sample size present greater challenges for neural architecture optimization than its CIFAR-10 counterpart. The dataset maintains the same image resolution (32 × 32 pixels) and color format as CIFAR-10 while substantially expanding taxonomic complexity, which has led to its adoption as a standard benchmark for evaluating hierarchical classification capabilities in both traditional CNNs and advanced architectures like Vision Transformers.

### 4.2. Experimental Setup

The evolutionary-based neural network architecture search method proposed in this paper, where DSNNs and corresponding modules are implemented using Pytorch v2.7.0, is one of the most widely used open-source frameworks in deep learning, proposed by Facebook. The experimental environment used is shown in Table 3.

The algorithm proposed in this paper is implemented using the PyTorch framework. SpikingJelly is employed to implement SNN [47]. To verify the proposed method, an Nvidia GeForce GTX 1070Ti GPU is used to conduct search and evaluation on the CIFAR-10 and CIFAR-100 datasets. When searching on the datasets, this paper follows the common practice of NAS methods, randomly taking out 80% of the original training set for training, while using the remaining 20% as a validation set to calculate the classification error.

The high computational cost of DSNNs is primarily driven by several key factors, including the following.

- Massive Network Training Requirements: DSNNs necessitate extensive training of numerous network architectures, each requiring independent training and validation processes. This large-scale training significantly escalates computational demands, particularly when dealing with complex architectures and large datasets.
- Complexity of the Search Space: The search space for DSNNs is vast and intricate, encompassing a multitude of potential network structures, layer configurations, and connection patterns. Conducting a thorough search within this complex space to identify the optimal architecture is computationally intensive.
- High Cost of Independent Evaluation: Each candidate network structure in DSNNs must be evaluated independently, involving separate training from scratch. This independent evaluation process, especially when dealing with large populations of candidate structures, dramatically increases computational overhead.
- Lack of Gradient Information: DSNNs often rely on evolutionary or heuristic methods that do not leverage gradient information. Unlike gradient-based optimization methods, which can guide the search more efficiently, these methods require extensive exploration of the search space, leading to higher computational costs.
- Hardware Resource Limitations: The computational requirements of DSNNs are substantial, often necessitating the use of high-performance hardware such as GPUs or TPUs. The need for extensive computational resources, especially for large-scale training and evaluation, poses significant challenges and contributes to the overall cost.

These factors collectively contribute to the high computational cost of DSNNs, making it a critical challenge in their development and application. Therefore, in the population initialization stage, the population size *N* is set to 20, the number of training iterations (Max_Gen) is 20, the number of membranes (*G*) is 4, and the batch size is 256. Each generation is trained for 100 epochs using the Adam optimizer until the best-performing network model is selected from the final generation. Parameters and their justifications used in the evolutionary membrane algorithm are shown in Table 4.

In the proposed algorihtm, the process can be divided into two stages, including the supernet training stage based on weight-sharing strategy and the sub-network search stage based on the evolutionary membrane algorithm after the supernet training is completed. During the supernet training stage, for the candidate operation blocks of the candidate layers in the supernet, this paper adopts a uniform sampling method to randomly select a candidate operation block on each mini-batch to form a single-path network for training. After the supernet training is completed, the evolutionary search stage begins.

### 4.3. Experimental Results and Discussion

Based on the parameter settings in Section 4.2 and the search space designed in Section 3.1, the optimal neural network searched under the experimental environment shown in Table 3 was tested on the CIFAR-10 and CIFAR-100 datasets. The experimental results are compared with those of mainstream manually designed deep neural networks and neural architecture search methods, as shown in Table 5 and Table 6.

In Table 5, the network searched by the proposed algorithm achieved a model accuracy of 94.77% on the CIFAR-10 dataset. The network models VGG-net [48], ResNet-101 [49], MobileNetV2 [50], and CIFARNet-Fang [51] are current mainstream manually designed network architectures. Compared with these manually designed network architectures, the method proposed in this paper has significant advantages in model accuracy. In addition to the comparisons mentioned above, the proposed algorithm was also compared with mainstream neural architecture search methods based on evolutionary algorithms. Specifically, Genetic-CNN [52] and AutoSNN [44] achieved model accuracies of 94.61% and 93.15%, respectively, on the CIFAR-10 dataset. The proposed algorithm outperforms these methods in terms of model accuracy. Although both methods adopt the same weight-sharing-based supernet training strategy, the evolutionary membrane strategy designed in this paper can express more diverse and distinct population objects, making it less likely to fall into local optima and thus finding more optimal network structures.

Compared with the network architecture searched on CIFAR-10, due to the higher number of classes and the increased complexity of the CIFAR-100 dataset, the network architecture requires more modules with attention mechanisms to enhance model accuracy, while relatively fewer identity mapping modules are used. This results in a certain increase in model parameters.

To further verify the effectiveness of the method proposed in this paper on more complex datasets, relevant experiments and result comparisons were conducted on the CIFAR-100 dataset. As shown in Table 6, compared with manually designed network structures, the network searched by the method proposed in this paper demonstrates superior performance in terms of model accuracy. When compared with evolutionary-based neural architecture search methods, the proposed method achieved improvements in model accuracy of 77.83% over Genetic-CNN [52], and AutoSNN [44].

### 4.4. Discussion

The weight-sharing mechanism of the supernet significantly reduces search time and computational resource consumption. By sampling and evaluating sub-network architectures within the supernet, the need for training each candidate sub-network from scratch is eliminated. The membrane computing framework allows flexible adjustment of the number of membranes and communication mechanisms to adapt to different search spaces and task requirements. Crossover and mutation operations are employed during the evolutionary search process based on the current search state to optimize search efficiency.

Weight sharing and membrane computing in DSNN provide substantial benefits but also introduce notable trade-offs. Weight sharing reduces computational costs by allowing multiple architectures to share a common set of weights, thus avoiding independent training for each architecture. However, shared weights may not be optimal for all architectures, potentially leading to suboptimal performance. Additionally, managing and synchronizing parallel tasks in membrane computing can be complex, and the benefits of parallel processing are limited by hardware resources. Addressing these trade-offs is essential for achieving robust and efficient neural architecture search.

From the experiments, it can be seen that for the one-shot neural architecture search method, searching for sub-networks within a supernet that contains a vast discrete search space is one of the key factors affecting the performance of the final searched network model. The evolutionary membrane algorithm combined with the weight-sharing strategy has the advantage of maintaining population diversity, which provides a guarantee of searching for well-performing network structures. The incorporation of the evolutionary membrane algorithm with the crossover rule and mutation rule significantly enhances the population updating ability, making it easier to escape local optima and find more optimal network structures. Multiple experiments validated the robustness of the proposed algorithm. It consistently identified well-performing sub-network architectures across different datasets and supernet configurations.

## 5. Conclusions

This paper makes novel contributions to the field of deep spiking neural networks (DSNNs) through the proposal of an evolutionary membrane algorithm and innovative concepts for spiking neural architecture search. Specifically, it focuses on tackling the existing challenges and complexities in DSNN architecture search. This study aims to effectively integrate DSNN architecture search methods into the design of evolutionary membrane algorithms in this relatively underexplored domain, thereby undertaking meaningful explorations and attempts. To tackle challenges like the high complexity of manual design, subpar model accuracy, and the incompatibility of traditional gradient-based search methods with DSNNs, we propose an evolutionary-based DSNN architecture search approach. Extensive experiments were conducted to validate the effectiveness of the proposed method, and comparisons with state-of-the-art approaches on the CIFAR-10 and CIFAR-100 datasets revealed improvements in model accuracy.

Despite these achievements, there are still some limitations in this research. For instance, the DSNN architecture search methods are currently implemented under ideal conditions due to the constraints of existing computing hardware. However, with the advancement and widespread adoption of neuromorphic computing hardware, the algorithmic ideas and methods proposed in this paper hold great potential for deployment and practical application. On the other hand, while supernet sampling training methods in DSNNs are crucial for efficient neural architecture search, they also introduce potential challenges that need to be carefully managed. These challenges include sampling bias, limitations of weight sharing, and inconsistencies between training and evaluation. Future efforts will concentrate on four fronts: (i) devising more efficient training techniques, (ii) exploring distributed training strategies, (iii) conducting extensive evaluations across diverse datasets, and (iv) integrating biologically plausible learning rules to boost the robustness, scalability, and real-world applicability of DSNNs. Moreover, acknowledging that real-world deployments frequently encounter class-imbalanced data, we will extend our algorithm to long-tailed (LT) variants of CIFAR and ImageNet-LT and adopt recall, F1 score, and AUC to rigorously assess its robustness under skewed distributions.

## Figures and Tables

**Table 1:** Comparison of deep neural networks (DNNs) and spiking neural networks (SNNs).

Feature	DNNs	SNNs
Neuron Model	Artificial neurons with weighted sums and activation functions (e.g., ReLU, sigmoid)	Biological neurons with spiking events and threshold-based firing
Temporal Dynamics	Static processing without explicit temporal dynamics	Inherently temporal with spike timing and event-driven processing
Energy Efficiency	High computational and energy requirements, especially on GPUs/CPU	More energy-efficient due to sparse and event-driven nature
Training Complexity	Backpropagation and gradient descent, well-established but computationally intensive	More complex due to non-differentiable spike events, often requiring surrogate gradients
Architecture Design	Dense layers, convolutional layers, recurrent layers (e.g., CNNs, RNNs)	Specialized architectures leveraging spiking dynamics (e.g., SCNs, SRNs)
Application Domains	Image recognition, natural language processing, reinforcement learning	Temporal data, gesture recognition, auditory processing, real-time sensor data
Scalability	Highly scalable to very large models (e.g., Transformers, GPT series)	Scalability is an ongoing research area, with recent advances in scaling up SNNs
Biological Plausibility	Less biologically plausible compared to SNNs	More biologically plausible, closely mimicking brain function
Advantages	- Well-established training methods - Broad range of applications - Extensive tools and frameworks available	- Energy-efficient - Suitable for temporal data - Biologically plausible
Challenges	- High computational and energy costs - Limited in temporal data processing	- Complex training - Design complexity - Limited tools and frameworks

**Table 2:** List of classes in the CIFAR-100.

Superclass	Classes
aquatic mammals	beaver, dolphin, otter, seal, whale
fish	aquarium fish, flatfish, ray, shark, trout
flowers	orchids, poppies, roses, sunflowers, tulips
food containers	bottles, bowls, cans, cups, plates
fruit and vegetables	apples, mushrooms, oranges, pears, sweet peppers
household electrical devices	clock, computer keyboard, lamp, telephone, television
household furniture	bed, chair, couch, table, wardrobe
insects	bee, beetle, butterfly, caterpillar, cockroach
large carnivores	bear, leopard, lion, tiger, wolf
large man-made outdoor things	bridge, castle, house, road, skyscraper
large natural outdoor scenes	cloud, forest, mountain, plain, sea
large omnivores and herbivores	camel, cattle, chimpanzee, elephant, kangaroo
medium-sized mammals	fox, porcupine, possum, raccoon, skunk
non-insect invertebrates	crab, lobster, snail, spider, worm
people	baby, boy, girl, man, woman
reptiles	crocodile, dinosaur, lizard, snake, turtle
small mammals	hamster, mouse, rabbit, shrew, squirrel
trees	maple, oak, palm, pine, willow
vehicles 1	bicycle, bus, motorcycle, pickup truck, train
vehicles 2	lawn-mower, rocket, streetcar, tank, tractor

**Table 3:** The experimental environment.

Component	Model
CPU	Dual Intel Xeon Platinum 8160 processor (33 M cache, 2.10 GHz)
	Two Xeon CPUs containing 48 parallel cores and 96 threads
Memory	160 GB
GPU	Nvidia GeForce GTX 1070Ti
Storage	1 TB

**Table 4:** Parameters and their justifications used in the evolutionary membrane algorithm.

Parameter	Value	Justification
Population Size (N)	20	Ensures sufficient diversity and avoids premature convergence while keeping computational cost manageable.
Number of Training Iterations (Max_Gen)	20	Provides enough generations for convergence without excessive computational time.
Number of Membranes (G)	4	Allows structured exploration of the search space without becoming overly complex.
Batch Size	256	Ensures efficient training while keeping memory usage within practical limits.
Mutation Rate	0.1	Introduces moderate genetic diversity to escape local optima and explore new regions.
Crossover Rate	0.7	Promotes combination of good traits from different parents, leading to better offspring solutions.
Early Stopping Threshold	0.05	Terminates evaluation of underperforming architectures early to save computational resources.
Learning Rate	0.001	Ensures stable and effective training of neural networks, chosen based on standard practices.

**Table 5:** Comparison of experimental results on the CIFAR-10 dataset.

Network Model	Search Method	Accuracy (%)
VGG-net [48]	Manual	93.34
ResNet-101 [49]	Manual	93.57
MobileNetV2 [50]	Manual	94.56
CIFARNet-Fang [51]	Manual	93.15
Genetic-CNN [52]	EA	94.61
AutoSNN [44]	EA	93.15
**Our Method**	EA	**94.77**

**Table 6:** Comparison of experimental results on the CIFAR-100 dataset.

Network Model	Search Method	Accuracy (%)
VGG-net [48]	Manual	67.95
ResNet-101 [49]	Manual	74.84
MobileNetV2 [50]	Manual	77.09
CIFARNet-Fang [51]	Manual	66.83
Genetic-CNN [52]	EA	74.88
AutoSNN [44]	EA	69.16
**Our Method**	EA	**77.83**

## Data Availability

The original contributions presented in the study are included in the article, further inquiries can be directed to the corresponding authors.
